# Depth-resolved birefringence imaging of collagen fiber organization in the human oral mucosa *in vivo*

**DOI:** 10.1364/BOE.10.001942

**Published:** 2019-03-22

**Authors:** Julia Walther, Qingyun Li, Martin Villiger, Camile S. Farah, Edmund Koch, Karol Karnowski, David D. Sampson

**Affiliations:** 1 TU Dresden, Faculty of Medicine Carl Gustav Carus, Department of Medical Physics and Biomedical Engineering, 01307 Dresden, Germany; 2 TU Dresden, Faculty of Medicine Carl Gustav Carus, Anesthesiology and Intensive Care Medicine, Clinical Sensoring and Monitoring, 01307 Dresden, Germany; 3 Optical + Biomedical Engineering Laboratory, Department of Electrical, Electronic & Computer Engineering, The University of Western Australia, Perth, WA 6009, Australia; 4 Harvard Medical School and Massachusetts General Hospital, Wellman Center for Photomedicine, Boston, MA, USA; 5 UWA Dental School, The University of Western Australia, Perth, WA 6009, Australia; 6 Australian Centre for Oral Oncology Research and Education, Perth, WA 6009, Australia; 7 University of Surrey, Guilford, Surrey GU2 7XH, United Kingdom

## Abstract

Stromal collagen organization has been identified as a potential prognostic indicator in a variety of cancers and other diseases accompanied by fibrosis. Changes in the connective tissue are increasingly considered for grading dysplasia and progress of oral squamous cell carcinoma, investigated mainly *ex vivo* by histopathology. In this study, polarization-sensitive optical coherence tomography (PS-OCT) with local phase retardation imaging is used for the first time to visualize depth-resolved (i.e., local) birefringence of healthy human oral mucosa *in vivo*. Depth-resolved birefringence is shown to reveal the expected local collagen organization. To demonstrate proof-of-principle, 3D image stacks were acquired at labial and lingual locations of the oral mucosa, chosen as those most commonly affected by cancerous alterations. To enable an intuitive evaluation of the birefringence images suitable for clinical application, color depth-encoded *en-face* projections were generated. Compared to *en-face* views of intensity or conventional cumulative phase retardation, we show that this novel approach offers improved visualization of the mucosal connective tissue layer in general, and reveals the collagen fiber architecture in particular. This study provides the basis for future prospective pathological and comparative *in vivo* studies non-invasively assessing stromal changes in conspicuous and cancerous oral lesions at different stages.

## 1. Motivation

Oral squamous cell carcinoma (OSCC) is the most common malignant lesion of the oral cavity and is considered to be mostly curable in the case of early detection and treatment [1,2]. Progression to malignancy is primarily related to two components – the action of malignant epithelial cells and the reaction of the stroma (extracellular matrix) of the oral mucosa [3]. Whereas the epithelial component of OSCC has been the subject of many studies, the role of the extracellular matrix has more recently captured researchers’ attention [4–8]. Most studies have investigated stromal changes *ex vivo*, in particular, in collagen fiber organization (i.e., arrangement and density) by polarized light microscopy using Picrosirius red staining (PSR-POL) [4–9]. Whereas PSR-POL is an established method for visualizing collagen fibers in histopathological sections, due to its low cost and availability in most clinical laboratories, second harmonic generation (SHG) microscopy is the gold standard for assessing collagen properties in experimental research settings, because of its high resolution and nondestructive imaging of both stained and unstained tissues up to 200 µm thick, many times the thickness of standard 5-µm pathology sections. However, the link between reduced collagen organization and oral tumor progression has primarily been investigated by means of PSR-POL [4–8] and only occasionally by SHG microscopy [10,11]. PSR-POL has shown that the oral stroma undergoes a change in birefringence, because of altered arrangement and density of its collagen fibrils, predominantly caused by tumor invasion [8]. Well-differentiated OSCCs often show distinct deposits of collagen with higher birefringence around the tumor; whereas, moderately and poorly differentiated OSCCs show lower birefringence.

PSR-POL is limited to histology and SHG microscopy is challenging *in vivo* due to the limited field-of-view (FOV) and high magnification. As an interesting alternative, we propose polarization-sensitive optical coherence tomography (PS-OCT) for *in vivo* imaging of collagen fiber organization in the human oral mucosa, employing the intrinsic birefringence of collagen as the contrast mechanism, and combined with reconstructing the local, depth-resolved tissue birefringence. Even though optical coherence tomography (OCT) has inferior spatial resolution (1-20 µm lateral, 1-15 µm axial) than SHG microscopy (~0.5 µm lateral, 1-2 µm axial), it is attractive due to its large imaging depth of ~1 mm in soft tissue, wide FOV (from a few to tens of square millimeters), fast acquisition rates and workflows, with the potential for real-time imaging under *in vivo* conditions, suitable for clinical translation [12]. Conventional intensity-based OCT has previously been used for the detection of  oral  (pre- ) cancerous  lesions, in animal and human studies, to evaluate *in vivo* oral dysplasia and malignancies [13–16], but without investigating the polarization properties of the oral mucosa.

By extending OCT to measuring the polarization state of the light returning from the sample, additional tissue-specific contrast, such as birefringence of aligned fibrous structures, is obtained [17,18]. In most PS-OCT studies in biomedical and clinical research, including previous studies on healthy and pathologically altered oral mucosa in animal models [19] and humans [20–25], images showing cumulative (phase) retardation has been reported. Some studies have started to improve this by presenting *en-face* parametric maps of birefringence averaged over appreciable fractions of a millimeter, with encouraging results [26,27]. The problem is that cumulative retardation is difficult to interpret locally, on a depth-resolved basis, especially in tissues consisting of several layers with different values of birefringence. To address this, several different approaches, based on determining localized Jones matrices from depth-localized retardation measurements, have been introduced, considerably improving interpretation of birefringent tissue structures [28,29]. Since the local Jones matrix measurement is vulnerable to low SNR, averaging techniques have been introduced, e.g., based on the coherent Jones formalism [30–34] or the Mueller formalism [35–40]. Focusing on collagen assessment, local birefringence (henceforth, referred to as depth-resolved birefringence) imaging was performed using local retardation reconstruction methods for eye [32,41], skin [33,36] and breast [37] imaging.

To the best of our knowledge, for the first time, we visualize non-invasively the stromal collagen organization in healthy oral soft tissue *in vivo* with images of depth-resolved birefringence, reconstructed from measurements with PS-OCT. Moreover, for the first time, healthy oral collagen structures are visualized using a novel *en-face* color-based depth-encoded projection of depth-resolved birefringence obtained using PS-OCT. The current realization has an FOV of 2.1 mm × 2.1 mm, images collagen at depths up to 400 µm, and conveys all essential information conveniently in a single image. Our method and results suggest the way forward for further studies assessing malignant stromal alterations in oral cancer.

## 2. Methods

### 2.1 Experimental setup

**Fig. 1:**
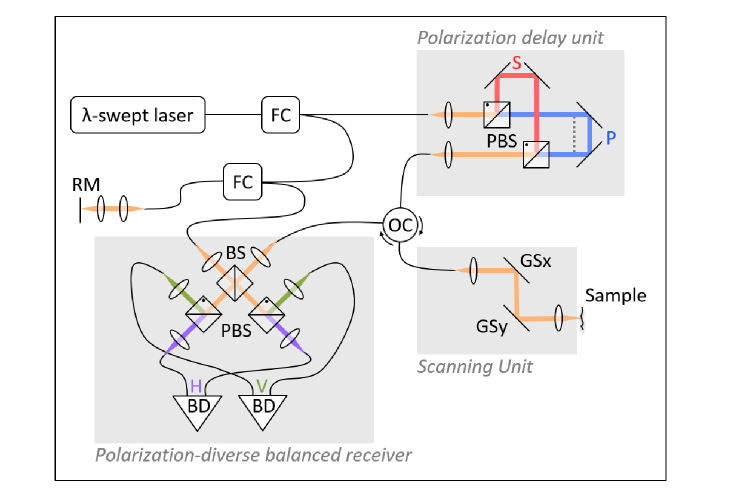
PS-OCT system with standard scanner head modified from [37] for imaging the oral mucosa of the anterior human oral cavity *in vivo*. The system contains a swept laser source, a fiber-based interferometer including a polarization delay unit, a scanning unit for 2D beam deflection, a reference arm and a polarization-diverse balanced receiver. FC, fiber coupler; OC, optical circulator; RM, reference mirror; BS, beamsplitter; PBS, polarizing beamsplitter; S,P, orthogonal input polarization states; BD, balanced detectors; H,V, horizontal and vertical polarization state channels; GS, galvanometer scanner.

The PS-OCT system setup was based on a wavelength-swept light source (AXP50125-6, Axsun, USA) centered at λ = 1310 nm with Δλ = 110 nm and a modified fiber-coupled Mach-Zehnder interferometer (Fig. 1

), and has been described in detail recently [37]. Imaging at 1300 nm wavelength provides deeper penetration than at 800 nm but a detailed comparison of wavelengths has not been made in the oral mucosa [42–44].

Briefly, a free-space polarization delay unit (PDU) was placed in the sample arm to generate two orthogonal linear polarization states with different delays (passive polarization multiplexing) [45–47]. After recombination of the light from sample and reference arms using a non-polarizing beamsplitter, its two outputs were further split into orthogonal polarization states by polarization beamsplitters (PBS) in each path and separately detected with balanced detectors (PDB460C-AC, Thorlabs Inc., USA), often referred to as a polarization-diverse balanced receiver. The system acquisition rate was electronically adapted using the frequency-doubled sampling clock of the swept source to achieve an adequate imaging depth range (similar to [47]) for recording of the depth-multiplexed polarization states. A trigger generated by a fiber Bragg grating is used to remove the timing jitter issue in [37]. A standard OCT scanning unit, consisting of a fiber collimator (F220APC-1310, Thorlabs Inc., USA), a pair of galvanometer scanners (GVS002, Thorlabs Inc, USA) and a scanning lens (LSM02, Thorlabs Inc., USA), was used for imaging. A set of 1000 × 1000 A-scans was acquired over an area of (2.1 × 2.1) mm^2^ at an A-scan rate of 50 kHz. The measured lateral and axial resolutions are 12 µm and 14 µm, respectively; thereby, the FOV was oversampled at six samples per resolution element.

### 2.2 Anatomical and histological background

For the interpretation of the subsequent imaging results of non-birefringent and birefringent oral structures, the histological and anatomical background is briefly given below. In general, the human oral mucosa consists of stratified squamous epithelium (EP) and an underlying connective tissue distinguished into lamina propria (LP) and submucosa. In turn, the lamina propria is divided into two layers: the superficial papillary layer (PL), associated with an irregular convoluted interface consisting of finger-like projections of connective tissue extending into the lower aspect of the epithelium; and the deeper reticular layer (RL) of grid-like tight connective tissue. Whereas the collagen fibers of the PL are thin and loosely arranged, the RL contains closely packed and net-like arranged bundles of collagen fibers which tend to lie in a plane parallel to the surface.

**Fig. 2:**
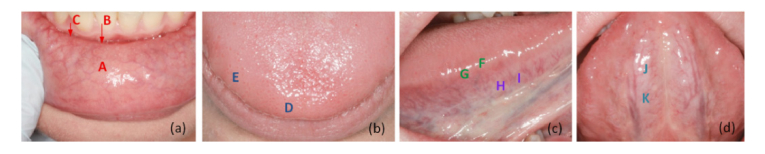
Measurement points within the anterior oral cavity for representative polarization-sensitive OCT imaging of the oral mucosa. (a) Position A: labial oral mucosa of inner lower lip; Positions B and C: vestibular (mucolabial fold) mucosa; (b) Positions D and E: tip of the dorsal tongue; (c) Positions F and G: lateral dorsal tongue, and Positions H and I: transition of dorsal to ventral lateral tongue; (d) Positions J and K: ventral tongue.

For this proof-of-principle study, representative mucosal structures of the readily accessible anterior oral cavity of one volunteer (female 34 years, nonsmoker) were imaged by PS-OCT in contact mode with a standard scanner head in combination with a thick transparent optical quartz glass window. Multiple measurement points of the healthy non-altered oral mucosa were defined with advice from an experienced oral medicine specialist (Fig. 2

). The selection is based on highly accessible areas of the anterior oral cavity, which are statistically most frequently affected by alterations, such as the labial and lingual mucosa, as well as the floor of the mouth. In detail, the labial mucosa on the inner aspect of the lower lip (A) and the labial mucosa close to the vestibule (mucolabial fold) (B,C) were imaged. With regard to the tongue mucosa, the dorsal aspect at the tip of the tongue (D,E), the lateral aspect of the tongue (F,G), as well as the transition from dorsal to ventral tongue (H,I) were imaged. Finally, two locations of the ventral anterior tongue were imaged (J,K).

### 2.3 Image processing

**Fig. 3:**
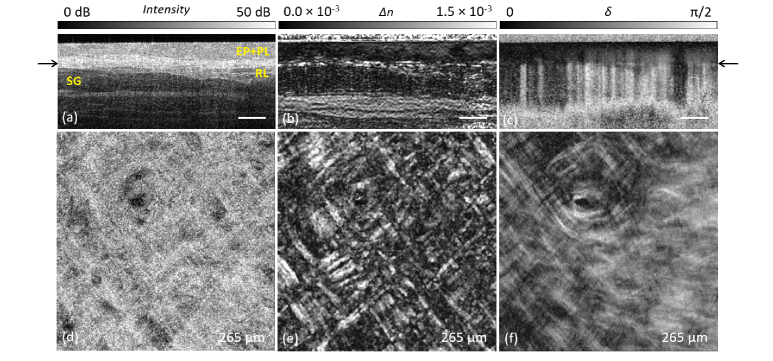
(a,b,c) Cross-sectional (B-scan) images of the labial oral mucosa by PS-OCT. (a) Intensity image showing the epithelium (EP), papillary layer (PL) and the the dense, fibrous reticular layer (RL). The border between the EP and PL cannot be readily located on the basis of the intensity image. Minor salivary glands (SG) are below the RL. (b) Depth-resolved birefringence (*Δn*) revealing highly aligned collagen fibers within the RL immediately below and parallel to the EP; (c) Cumulative phase retardation (*δ*); Images are scaled in depth using refractive index of n = 1.4 [48]. Scale bar: 300 µm. (d,e,f) *En-face* projections (single slices; imaged area: 2.1 mm × 2.1 mm) of the corresponding data set for (d) the intensity signal (dynamic range 30 dB); (e) the depth-resolved birefringence (*Δn* range 0.0 × 10^−3^ - 1.5 × 10^−3^); (f) the cumulative phase retardation (δ range 0 to *π/2*) at a depth of 265 µm below the surface (black arrows in (a) and (c)).

Image processing and reconstruction of tissue birefringence is mathematically described in detail in [37]. Briefly, birefringence is computed by constructing Mueller-Jones matrices from measured Jones matrices with subsequent spatial filtering (3D Gaussian filter with an axial and lateral FWHM corresponding to twice the axial and lateral resolutions, respectively) and extraction of the local phase retardation by a differential Mueller matrix formalism [35,37]. Of particular relevance for the assessment of the collagen fiber organization is the generation of *en-face* projections briefly described below. A typical example of cross-sectional PS-OCT imaging of *in vivo* human labial oral mucosa (Fig. 2(a), position A) of the inner side of the lower lip is presented in Fig. 3

showing: (a) the backscattering intensity-based structural information; (b) depth-resolved tissue birefringence by means of the local phase retardation; and (c) the cumulative phase retardation. Regarding the structural intensity signal (Fig. 3(a)), the epithelium (EP) with the adjacent papillary layer (PL, upper part of the oral connective tissue) and subsequent reticular layer (RL) are visible. Beyond that, minor salivary glands (SG) can occasionally be found. Considering the birefringence information, the benefit of calculating the local phase retardation and reconstructing the depth-resolved birefringence (Fig. 3(b)) becomes apparent through the greatly improved visualization of highly aligned collagen fibers, running almost parallel to the EP layer, which is difficult to identify from the cumulative phase retardation (Fig. 3(c)). There, vertical lines of higher retardation commencing in the RL result from the cumulative measurement, where the polarization change of the incoming light due to aligned collagen fibers in the RL is retained in the cumulative retardation whilst the light travels through the subsequent non-birefringent salivary gland. To gain a better view of the collagen fiber organization, single *en-face* slices were generated from the same data set for (d) the intensity signal, (e) the depth-resolved birefringence and (f) the cumulative phase retardation, visualizing single projection views within the RL at a depth of 265 µm below the surface (black arrows in Figs. 3(a) and (c)).

Even with careful inspection of the cumulative retardation (Fig. 3(f)), the collagen fiber structure organization within the RL is more difficult to interpret due to the cumulative measurement. There, the connective tissue papillae within the overlaid PL already result in a low cumulative retardation, which is blurred across the entire width of the *en-face* slices over the whole measurement depth range. In addition to the collagen fiber layer above the salivary gland (SG), muscle structure can be identified by the strong birefringence signal at greater depth (Fig. 2(b)).

**Fig. 4:**
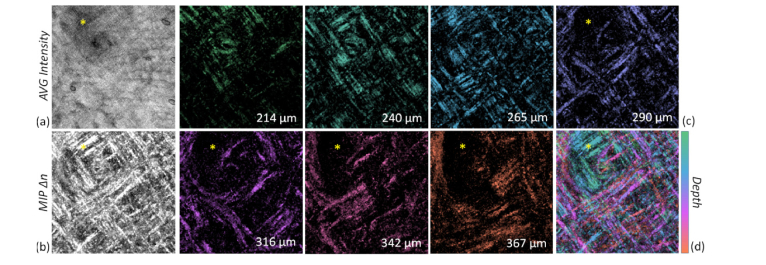
(a,b) Averaged (*AVG*) intensity and maximum intensity projection of the depth-resolved birefringence (*MIP Δn*) of N = 30 *en-face* slices within the RL at measurement point A in Fig. 2(a). (c) Corresponding color depth-encoded tissue birefringence (*∆n*) for representative depths within the absolute range 214-367 µm below the surface. (d) Resulting color depth-encoded birefringence using isoluminant colormap; Scaling: intensity 20 dB; tissue birefringence Δn 0.3 × 10^−3^ - 1.5 × 10^−3^. Salivary glands marked by asterisk. The imaged area corresponds to 2.1 mm × 2.1 mm.

We anticipate that it may be beneficial in future clinical applications to visualize the collagen organization in a single *en-face* projection, instead of a series of single *en-face* images. Thus, we reduced the 3D stack of local birefringence *∆n* voxels to a 2D image with color-encoded depth information. This visualization is shown for the stack of *en-face* slices of the preceding data set (Fig. 3) via the average intensity projection (Fig. 4(a)

, AVG intensity) as well as the maximum intensity projection of the local birefringence *∆n* (Fig. 4(b), MIP *∆n*) for the depth range of 214-367 µm below the surface. AVG intensity gave better contrast by inspection than MIP intensity. The depth range representing the RL is manually determined from the averaged birefringence and intensity signals of the detected 1000 × 1000 A-scans versus depth.

The 3D stack of *Δn en-face* slices is color-encoded to highlight the variation of collagen fiber alignment versus depth, as shown in Fig. 4(c). The maximum intensity projection of this color-encoded sequence of *Δn,* for the depth range containing the RL with tight collagen fiber content, was generated using the ‘Time Series Color Coder’ macro for Fiji software with a specifically designed isoluminant look-up table [49] and is presented in Fig. 4(d). The depth-resolved tissue birefringence is, thus, presented in a single color-depth-encoded *en-face* projection, which offers considerably enhanced contrast over the simple averaged (AVG) intensity signal. Additionally, the visibility of the grid-like collagen fiber network is greatly enhanced by means of simple MIP *Δn*.

## 3. Experimental results

The labial and lingual oral mucosa offer the highest level of collagen fiber organization in the healthy oral cavity and are, besides the floor of the mouth, the most susceptible areas to carcinogenic alterations. Therefore, these readily accessible regions were used in this proof-of-principle study of depth-resolved birefringence imaging of healthy human oral mucosa.

### 3.1 Labial oral mucosa

**Fig. 5:**
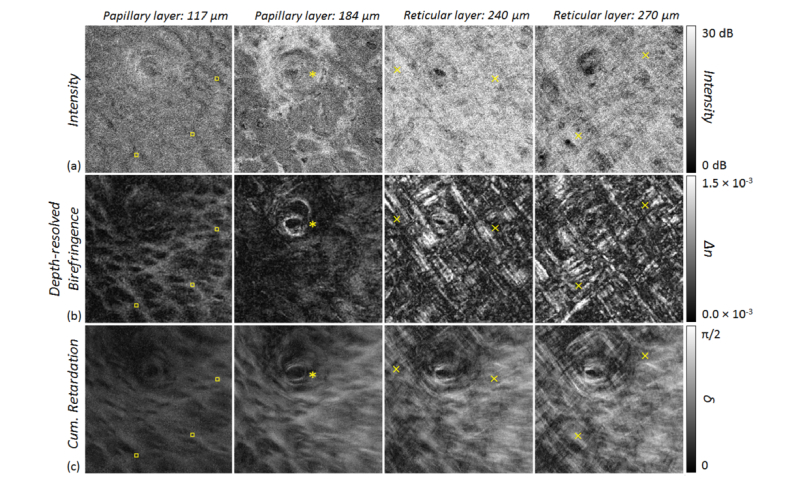
*En-face* projections (single slices) of the labial oral mucosa detected at position A in Fig. 2(a) and displayed via B-scans in Fig. 3. (a) Intensity; (b) depth-resolved birefringence (*Δn*); and (c) cumulative retardation (*δ*). Depth position 117 µm: papillary layer with epithelial rete ridges and connective tissue papillae showing birefringence due to stretched connective tissue papillae (squares). Depth position 184 µm: transition from PL to RL of the lamina propria with visible gland duct orifice (asterisk). Depths 240 µm and 270 µm: aligned collagen fibers within RL (cross signs). The imaged area corresponds to 2.1 mm × 2.1 mm.

Imaging results of the labial oral mucosa at measurement point A (Fig. 2), are presented as a series of *en-face* images showing the transition from epithelium (EP) to connective tissue layer (papillary and reticular layers, PL and RL) by means of the intensity signal and the depth-resolved birefringence, as well as the cumulative phase retardation (Fig. 5

). For this example, *en-face* slices were first extracted at depths of 117 µm and 184 µm below the surface for presenting the papillary layer, which contains finger-like connective tissue papillae extending beyond the average epithelial layer depth visible through their lower scattering as seen in the *en-face* intensity projection (squares in Fig. 5(a), depth 117 µm) and the noticeably birefringent circular structures in the *en-face* projection of the local (depth-resolved) birefringence (squares in Fig. 5(b), depth 117 µm). The gland duct orifice of a subsequent minor salivary gland is clearly identified by means of the circularly arranged collagen fiber bundles in the *en-face* slice (asterisk in Fig. 5(a,b), depth 184 µm), similarly imaged by cumulative retardation in a previous study [22]. Underneath, the lamina propria is visualized by its increased reflectivity (Fig. 5(a), depth 240 µm and 270 µm) caused by the highly backscattering connective tissue. The birefringent collagen network of the RL within the lamina propria is readily apparent as the grid-like tight arrangement of collagen fibers (cross signs in Fig. 5(b), depth 240 µm and 270 µm). Again, assessing the collagen fiber organization by means of the cumulative phase retardation is challenging because of slightly birefringent connective tissue papillae within the PL causing blurring of the retardation over the entire measurement range.

**Fig. 6:**
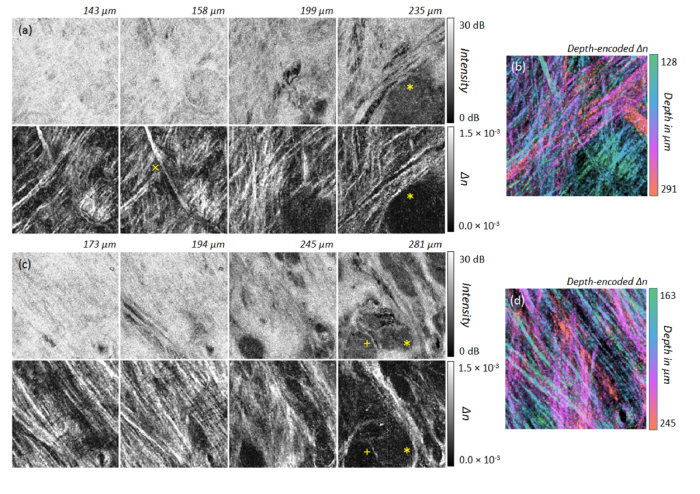
(a,c) Intensity and birefringence (*Δn*) at different depths of the reticular layer (RL) within the lamina propria of the inner side of the lower lip at measurement points in Fig. 2(a) labelled B (a) and C (c) presenting the vestibular mucosa (mucolabial fold). The alignment of collagen fibers within the RL and around salivary glands (asterisk) are more visible by means of local tissue birefringence than by intensity. The imaged area corresponds to 2.1 mm × 2.1 mm. (b,d) Corresponding color depth-encoded depth-resolved birefringence. (b) Position B: N = 33 *en-face* slices; *Δn*: 0.5 × 10^−3^ – 1.5 × 10^−3^. (d) Position C: N = 17 *en-face* slices; *Δn*: 0.5 × 10^−3^ – 1.5 × 10^−3^.

Additionally, a series of *en-face* images of the labial oral mucosa at measurement point B and C (Fig. 2), containing the intensity signal and the depth-resolved birefringence, are presented below (Fig. 6

). As expected, the alveolar mucosa close to the vestibular fold exhibits dense fibrous connective tissue with parallel aligned collagen fibers, which can be readily identified by the *en-face* projections of the local birefringence. Furthermore, minor salivary glands are found at larger depths presenting as low scattering regions (asterisk in Fig. 6(a), 6(c)). Apart from the birefringent structure indicated by the cross symbol in Fig. 6(a), collagen fibers are highly aligned. Additionally, a capsule of connective tissue surrounding the salivary gland is clearly visible in the depth-resolved birefringence projection (asterisk in Fig. 6(a), 6(c)). The septae of connective tissue originating from the capsule can be also identified (plus sign in Fig. 6(c)). Color depth-encoded *en-face *projections of the depth-resolved birefringence provide single images revealing the collagen fiber organization throughout the projected tissue depth range (Fig. 6(b), 6(d)).

### 3.2 Lingual oral mucosa

**Fig. 7:**
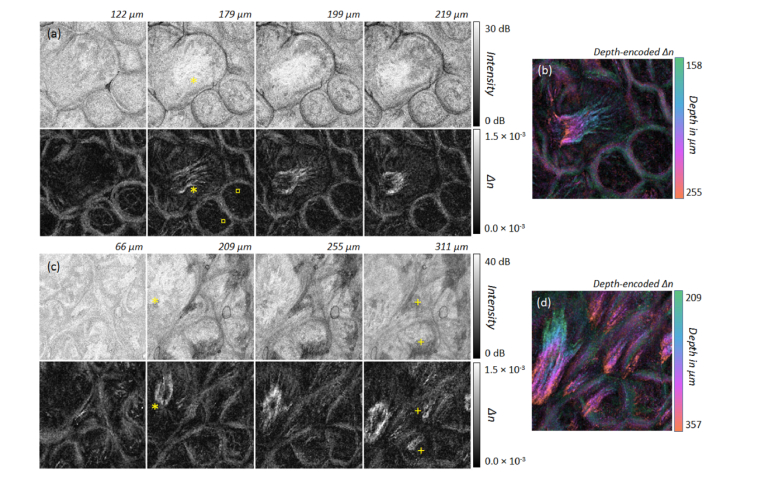
(a,c) Intensity and birefringence (*Δn*) at different depths of the dorsal tongue at measurement points in Fig. 2(b) labelled D (a) and E (c). The birefringence of the connective tissue core of the lingual papillae (filiform and fungiform papillae) is shown. The imaged area corresponds to 2.1 mm × 2.1 mm. (b,d) Corresponding color depth-encoded depth-resolved birefringence. (b) Position D: N = 20 *en-face* slices; *Δn*: 0.2 × 10^−3^ – 1.2 × 10^−3^. (d) Position E: N = 30 *en-face* slices; *Δn*: 0.1 × 10^−3^ – 1.5 × 10^−3^.

Malignant alterations are frequently observed at rapidly changing transitions between different epithelial types. Therefore, the dorsal side of the volunteer’s tongue was imaged *in vivo* at the tip and the outer third of the tongue body (Fig. 2(b), positions D and E). The dorsal side of the tongue is covered by a functionally masticatory mucosa with different types of lingual papillae. Images of the distinctive specialized oral mucosa, containing mainly filiform but also fungiform papillae, are presented for the tip of the tongue (Fig. 7(a)

, position D) and for the lateral side of the dorsal tongue (Fig. 7(c), position E).

In each example, a large papilla with a dominant highly reflecting connective core containing a collagen fiber bundle can be identified, due to the non-keratinized epithelium, as an indicator for a fungiform papillae (asterisk in Fig. 7(a), 7(c)). In addition, smaller filiform papillae (plus signs in Fig. 7(c)) are the most numerous lingual papillae with a keratin-containing epithelium and were imaged from the side (due to the contact mode measurement) so that the keratinized tip is not disruptive in visualizing the underlying structures. The two small papillae at the lower right side of the *en-face* projections (squares in Fig. 7(a)) exhibit a circular shape in combination with a backscattering signal from larger depth but no highly reflecting connective tissue core with corresponding birefringent signal. The reason for the weak birefringent contrast of the border of each papilla could be due to the orientation and/or the amount of aligned collagen fibers surrounding the papillae.

**Fig. 8:**
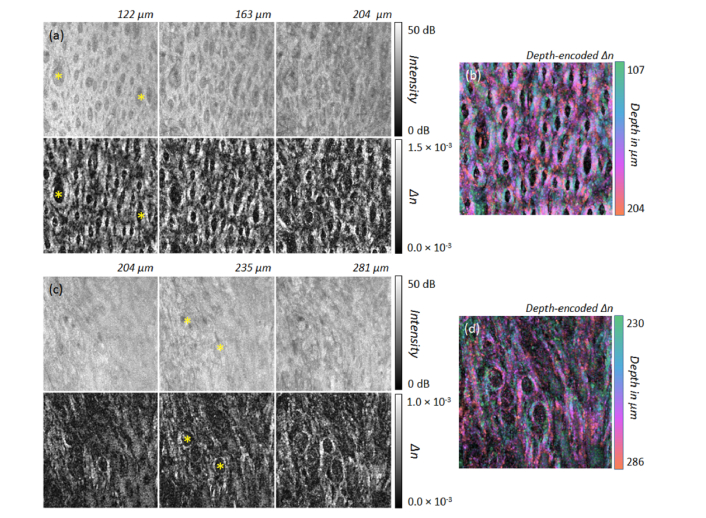
(a,c) Intensity and birefringence (*Δn*) at different depths of the transition region from dorsal to lateral tongue at measurement points in Fig. 2(c) labelled F (a) and G (c). The higher birefringence of aligned collagen fibers surrounding small lingual papillae can be seen. The imaged area corresponds to 2.1 mm × 2.1 mm. (b,d) Corresponding color depth-coded depth-resolved birefringence. (b) Position F: N = 20 *en-face* slices; *Δn*: 0.2 × 10^−3^ – 1.5 × 10^−3^. (d) Position G: N = 12 *en-face* slices; *Δn: *0.1 × 10^−3^ – 1.0 × 10^−3^.

Progressing towards the posterior lateral border of the tongue (points F and G in Fig. 2(c)), much smaller non-keratinized papillae become visible (asterisks in Fig. 8(a), 8(c)

). The filiform papillae do not have a significant connective tissue core detectable with PS-OCT but instead collagen fibers surround these small papillae. Comparing the measurements slightly closer to the dorsal side (position F in Fig. 2(c)) with those closer to the lateral tongue (position G in Fig. 2(c)), fewer papillae and lower birefringence due to a significantly lower content of thick aligned collagen fibers can be noted for the more lateral position (Fig. 8(c)).

**Fig. 9:**
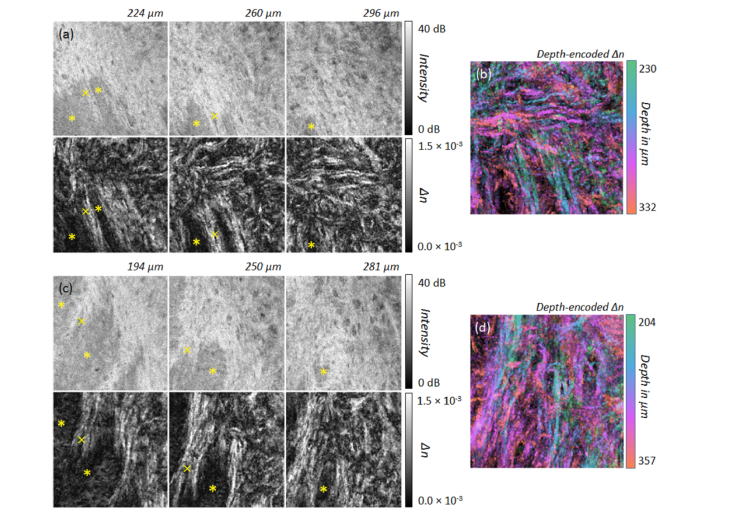
(a,c) Intensity and birefringence (*Δn*) at different depths of the transition from dorsal to ventral tongue at measurement points in Fig. 2(c) labelled H (a) and I (c). The imaged area corresponds to 2.1 mm × 2.1 mm. (b,d) Corresponding color depth-encoded depth-resolved birefringence. (b) Position H: N = 21 *en-face* slices; *Δn*: 0.3 × 10^−3^ – 1.5 × 10^−3^. (d) Position I: N = 31 *en-face* slices; *Δn*: 0.3 × 10^−3^ – 1.5 × 10^−3^.

In contrast, the collagen content is increased towards the ventral tongue but shows a high variability in fiber orientation, especially at larger depths. The parts with no birefringence and less backscattering intensity (asterisk in Fig. 9(a), 9(c)

) correspond to regions of the upper connective tissue (lower part of the papillary layer) with less collagen content in comparison to the adjacent reticular layer with highly aligned and strongly reflecting collagen fibers (cross signs in Fig. 9(a), 9(c)).

**Fig. 10:**
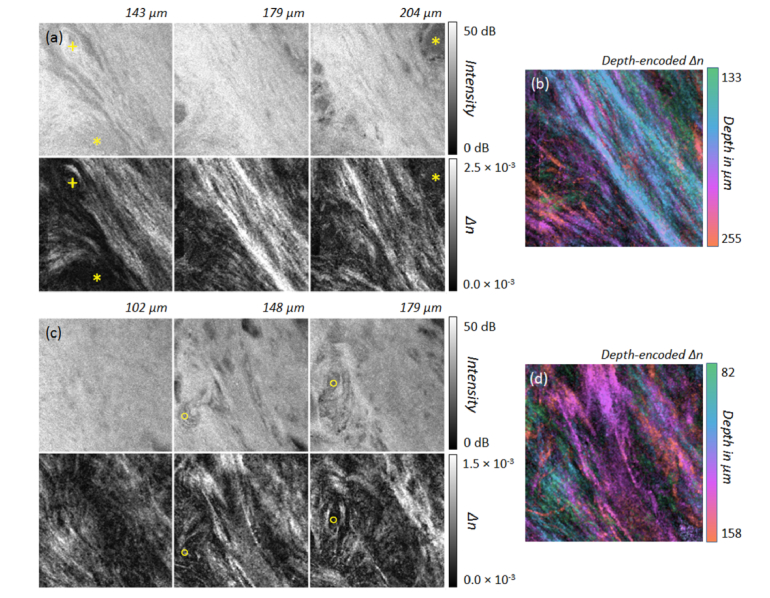
(a,c) Intensity and birefringence (*Δn*) at different depths of the ventral tongue at measurement points in Fig. 2(d) labelled J (a) and K (c). The imaged area corresponds to 2.1 mm × 2.1 mm. (b,d) Corresponding color depth-encoded depth-resolved birefringence. (b) Position J: N = 25 *en-face* slices; birefringence *Δn*: 0.3 × 10^−3^ – 2.5 × 10^−3^. (d) Position K: N = 16 *en-face* slices; *Δn*: 0.2 × 10^−3^ – 1.5 × 10^−3^.

For completeness, the ventral side of the tongue was imaged (positions J and K Fig. 2(d)). The results cannot be interpreted in a straightforward manner on first examination. However, with the prior knowledge gained here, one can again clearly identify less birefringent regions belonging to the upper connective tissue (papillary layer, asterisks in Fig. 10(a)

). Furthermore, highly reflecting regions with lower birefringence are probably anchor points, where collagen orientation is almost parallel to the incident sample beam, with the consequence that only less or no birefringence is measurable (plus sign in Fig. 10(a)). Additionally, the circular structures in the projection of the depth-resolved birefringence (circles in Fig. 10(c)) are caused by connective tissue surrounding a larger blood vessel. In general, the central part of the ventral tongue (Fig. 2(d)) contains a high content of aligned collagen fibers resulting in strong contrast in the depth-resolved birefringence projection images.

## 4. Discussion and conclusion

In previous PS-OCT studies, the human oral mucosa has been evaluated based on cumulative phase retardation [20–24], which is considerably more difficult to interpret than depth-resolved birefringence, as exemplified in Fig. 3 in this manuscript [34,36]. Furthermore, we have demonstrated an approach to collapsing 3D volumetric local-birefringence image data into depth-encoded *en-face* parametric images. In particular, our results demonstrate the great potential of depth-resolved birefringence for visualization of collagen fiber organization within the upper connective tissue layer. In detail, the healthy reticular layer (RL) we investigated, with measured thickness in the range 100-200 µm, is well suited to be represented by a single 2D MIP image. The conditions for imaging aligned collagen fibers within the RL of the oral lamina propria by PS-OCT are optimal because the fiber orientation is mainly perpendicular to the incident sample beam, particularly when using a glass plate in contact mode, as in the present study. Further, we have shown this configuration leads to strong backscattering signals, with good SNR, relatively large phase retardation and strong local birefringence contrast. Even though our OCT system, with spatial resolution of 12-14 µm, cannot directly resolve the fibrillar collagen (mainly type I and III [50]) within the reticular layer, the larger fiber composite structures it forms can be detected by the polarization-sensitive measurement with lateral oversampling (at six samples per resolution element), which allows for the effective depiction of the collagen fiber arrangement. Moreover, structures linked to aligned collagen fibers, such as gland duct orifices and subsequent minor salivary glands, can be identified. We additionally note that depth-resolved birefringence measured by PS-OCT is sensitive to the sub-resolution, even sub-wavelength, structures not spatially resolved, and therein lies one its key advantages as a source of additional contrast.

*En-face* projections of the 3D PS-OCT data set provide new insights into the collagen fiber organization of the oral mucosa, especially in comparison to common PS-OCT cross-sectional (B-scan) imaging and *ex vivo* PSR-POL, which is conventionally based on cross-sectioning in depth. For histological biopsy examination of thin tissue slices by means of PSR-POL, it is challenging to assess the collagen fiber organization as cross-sections typically cut across the collagen fiber plane. Thus, depth-resolved birefringence presented as *en-face* images could provide an alternative non-invasive means to access information on the connective tissue of the oral mucosa during prospective preoperative examinations. At the very least, PS-OCT could serve as an adjunct to routine histopathology for studying stromal changes at the invading front of the tumor island, which, in turn, could help to develop a grading system for tumor behavior/progression *in vivo*. For *in vivo* imaging, a hand-held device will be necessary, with rigid or fiber-based endoscopic head [12,51,52], combined with a sufficiently high scan rate to avoid motion artifacts. Additionally, the combination of optical coherence angiography (OCA) [51,53] revealing microvascular anatomy with PS-OCT may increase the potential for staging of oral tumor invasion in advanced cancerous lesions non-invasively.

With regard to oral squamous cell carcinoma in particular, previous studies using polarized light microscopy have shown that distinct deposits of collagen with high birefringence, mainly around the tumor islands (neoplastic epithelial cells/islands), are present in well-differentiated lesions [4–8,54]. The reason for the strong birefringence could be newly formed thick bands of collagen fibers consisting of densely packed fibrils [4,55,56]. In contrast, in moderately and poorly differentiated squamous cell carcinoma, the collagen fibers were reticular fibrillary and more disorganized. Thus, the extent to which invasion in oral squamous cell carcinoma may be assessed by depth-resolved birefringence using PS-OCT remains to be investigated in future studies. In general terms, the motivation is the improved distinction between the tumor island and the surrounding healthy collagen network within the thin RL and, consequently, the better evaluation of the tumor border as a possible marker for tumor progression *in vivo*. For this, the depth range used for the color-encoded MIP must be chosen appropriately depending on the tumor dimensions and the nature of its transition to the adjacent healthy RL.

In summary, PS-OCT with depth-resolved birefringence based on a differential Mueller matrix formalism [35,36] was used for the first time for the visualization of the collagen content of the fibrous connective tissue layer of healthy human oral mucosa. In this study, we have imaged and assessed variations in collagen fiber alignment in the human labial and lingual oral mucosa *in vivo*, with the main observation of high birefringence within the lamina propria caused by a high level of collagen organization. Looking forwards, color depth-encoded *en-face* projections may offer a more intuitive way of viewing OCT data, with the aim to make clinical assessment of the scanned oral area more efficient. The results of this study motivate future pathological and comparative studies assessing cancer-induced stromal changes in the human oral connective tissue non-invasively by PS-OCT.

## References

[1] Rivera C. Venegas B., “Histological and molecular aspects of oral squamous cell carcinoma (Review),” Oncol. Lett. 8(1), 7–11 (2014). 24959211 10.3892/ol.2014.2103 PMC4063640

[2] Dissanayaka W. L. Pitiyage G. Kumarasiri P. V. R. Liyanage R. L. P. R. Dias K. D. Tilakaratne W. M., “Clinical and histopathologic parameters in survival of oral squamous cell carcinoma,” Oral Surg. Oral Med. Oral Pathol. Oral Radiol. 113(4), 518–525 (2012). 10.1016/j.oooo.2011.11.001 22668430

[3] Feller L. Lemmer J., “Oral squamous cell carcinoma: epidemiology, clinical presentation and treatment,” J. Cancer Ther. 3(4), 263–268 (2012). 10.4236/jct.2012.34037

[4] Aparna V. Charu S., “Evaluation of collagen in different grades of oral squamous cell carcinoma by using the Picrosirius red stain - a histochemical study,” J. Clin. Diagn. Res. 4(6), 3444–3449 (2010).

[5] Arun Gopinathan P. Kokila G. Jyothi M. Ananjan C. Pradeep L. Humaira Nazir S., “Study of collagen birefringence in different grades of oral squamous cell carcinoma using picrosirius red and polarized light microscopy,” Scientifica (Cairo) 2015, 802980 (2015). 10.1155/2015/802980 26587310 PMC4637505

[6] Kardam P. Mehendiratta M. Rehani S. Kumra M. Sahay K. Jain K., “Stromal fibers in oral squamous cell carcinoma: A possible new prognostic indicator?” J. Oral Maxillofac. Pathol. 20(3), 405–412 (2016). 10.4103/0973-029X.190913 27721605 PMC5051288

[7] John R. E. Murthy S., “Morphological analysis of collagen and elastic fibers in oral squamous cell carcinoma using special stains and comparison with Broder’s and Bryne’s grading systems,” Indian J. Dent. Res. 27(3), 242–248 (2016). 10.4103/0970-9290.186242 27411651

[8] Devendra A. Niranjan K. C. Swetha A. Kaveri H., “Histochemical analysis of collagen reorganization at the invasive front of oral squamous cell carcinoma tumors,” J. Investig. Clin. Dent. 9(1), e12283 (2018). 10.1111/jicd.12283 28714220

[9] Drifka C. R. Loeffler A. G. Mathewson K. Mehta G. Keikhosravi A. Liu Y. Lemancik S. Ricke W. A. Weber S. M. Kao W. J. Eliceiri K. W., “Comparison of picrosirius red staining with second harmonic generation imaging for the quantification of clinically relevant collagen fiber features in histopathology samples,” J. Histochem. Cytochem. 64(9), 519–529 (2016). 10.1369/0022155416659249 27449741 PMC5006137

[10] Shah A. T. Skala M. C., “Ex vivo label-free microscopy of head and neck cancer patient tissues,” Proc. SPIE 9329, 93292B (2015). 10.1117/12.2075583

[11] Chen W. S. Wang Y. Liu N. R. Zhang J. X. Chen R., “Multiphoton microscopic imaging of human normal and cancerous oesophagus tissue,” J. Microsc. 253(1), 79–82 (2014). 10.1111/jmi.12102 24236445

[12] Walther J. Schnabel C. Tetschke F. Rosenauer T. Golde J. Ebert N. Baumann M. Hannig C. Koch E., “*In vivo* imaging in the oral cavity by endoscopic optical coherence tomography,” J. Biomed. Opt. 23(7), 071207 (2018). 10.1117/1.JBO.23.7.071207 29500877

[13] Wilder-Smith P. Lee K. Guo S. Zhang J. Osann K. Chen Z. Messadi D., “In vivo diagnosis of oral dysplasia and malignancy using optical coherence tomography: Preliminary studies in 50 patients,” Lasers Surg. Med. 41(5), 353–357 (2009). 10.1002/lsm.20773 19533765 PMC2862682

[14] Wilder-Smith P. Jung W. G. Brenner M. Osann K. Beydoun H. Messadi D. Chen Z., “In vivo optical coherence tomography for the diagnosis of oral malignancy,” Lasers Surg. Med. 35(4), 269–275 (2004). 10.1002/lsm.20098 15493024

[15] Tomlins P. H. Adegun O. Hagi-Pavli E. Piper K. Bader D. Fortune F., “Scattering attenuation microscopy of oral epithelial dysplasia,” J. Biomed. Opt. 15(6), 066003 (2010). 10.1117/1.3505019 21198177

[16] Tsai M.-T. Lee C.-K. Lee H.-C. Chen H.-M. Chiang C.-P. Wang Y.-M. Yang C.-C., “Differentiating oral lesions in different carcinogenesis stages with optical coherence tomography,” J. Biomed. Opt. 14(4), 044028 (2009). 10.1117/1.3200936 19725739

[17] de Boer J. F. Hitzenberger C. K. Yasuno Y., “Polarization sensitive optical coherence tomography - a review [Invited],” Biomed. Opt. Express 8(3), 1838–1873 (2017). 10.1364/BOE.8.001838 28663869 PMC5480584

[18] Baumann B., “Polarization sensitive optical coherence tomography: a review of technology and applications,” Appl. Sci. (Basel) 7(5), 474 (2017). 10.3390/app7050474

[19] Kim K. H. Park B. H. Tu Y. Hasan T. Lee B. Li J. de Boer J. F., “Polarization-sensitive optical frequency domain imaging based on unpolarized light,” Opt. Express 19(2), 552–561 (2011). 10.1364/OE.19.000552 21263595

[20] Gladkova N. Kiseleva E. Robakidze N. Balalaeva I. Karabut M. Gubarkova E. Feldchtein F., “Evaluation of oral mucosa collagen condition with cross-polarization optical coherence tomography,” J. Biophotonics 6(4), 321–329 (2013). 10.1002/jbio.201200059 22764058

[21] Walther J. Golde J. Kirsten L. Tetschke F. Hempel F. Rosenauer T. Hannig C. Koch E., “*In vivo* imaging of human oral hard and soft tissues by polarization-sensitive optical coherence tomography,” J. Biomed. Opt. 22(12), 121717 (2017). 10.1117/1.JBO.22.12.121717 29264891

[22] Yoon Y. Jang W. H. Xiao P. Kim B. Wang T. Li Q. Lee J. Y. Chung E. Kim K. H., “In vivo wide-field reflectance/fluorescence imaging and polarization-sensitive optical coherence tomography of human oral cavity with a forward-viewing probe,” Biomed. Opt. Express 6(2), 524–535 (2015). 10.1364/BOE.6.000524 25780742 PMC4354576

[23] Lee A. M. F. Cahill L. Liu K. MacAulay C. Poh C. Lane P., “Wide-field *in vivo* oral OCT imaging,” Biomed. Opt. Express 6(7), 2664–2674 (2015). 10.1364/BOE.6.002664 26203389 PMC4505717

[24] Sharma P. Verma Y. Sahu K. Kumar S. Varma A. V. Kumawat J. Gupta P. K., “Human ex-vivo oral tissue imaging using spectral domain polarization sensitive optical coherence tomography,” Lasers Med. Sci. 32(1), 143–150 (2017). 10.1007/s10103-016-2096-3 27807650

[25] Kuranov R. Sapozhnikova V. Turchin I. Zagainova E. Gelikonov V. Kamensky V. Snopova L. Prodanetz N., “Complementary use of cross-polarization and standard OCT for differential diagnosis of pathological tissues,” Opt. Express 10(15), 707–713 (2002). 10.1364/OE.10.000707 19451924

[26] Chin L. Yang X. McLaughlin R. A. Noble P. B. Sampson D. D., “*En face* parametric imaging of tissue birefringence using polarization-sensitive optical coherence tomography,” J. Biomed. Opt. 18(6), 066005 (2013). 10.1117/1.JBO.18.6.066005 23733021

[27] Yang X. Chin L. Klyen B. R. Shavlakadze T. McLaughlin R. A. Grounds M. D. Sampson D. D., “Quantitative assessment of muscle damage in the mdx mouse model of Duchenne muscular dystrophy using polarization-sensitive optical coherence tomography,” J. Appl. Physiol. 115(9), 1393–1401 (2013). 10.1152/japplphysiol.00265.2013 23990241

[28] Makita S. Yamanari M. Yasuno Y., “Generalized Jones matrix optical coherence tomography: performance and local birefringence imaging,” Opt. Express 18(2), 854–876 (2010). 10.1364/OE.18.000854 20173907

[29] Todorović M. Jiao S. Wang L. V. Stoica G., “Determination of local polarization properties of biological samples in the presence of diattenuation by use of Mueller optical coherence tomography,” Opt. Lett. 29(20), 2402–2404 (2004). 10.1364/OL.29.002402 15532281

[30] Lim Y. Yamanari M. Fukuda S. Kaji Y. Kiuchi T. Miura M. Oshika T. Yasuno Y., “Birefringence measurement of cornea and anterior segment by office-based polarization-sensitive optical coherence tomography,” Biomed. Opt. Express 2(8), 2392–2402 (2011). 10.1364/BOE.2.002392 21833376 PMC3149537

[31] Ju M. J. Hong Y.-J. Makita S. Lim Y. Kurokawa K. Duan L. Miura M. Tang S. Yasuno Y., “Advanced multi-contrast Jones matrix optical coherence tomography for Doppler and polarization sensitive imaging,” Opt. Express 21(16), 19412–19436 (2013). 10.1364/OE.21.019412 23938857

[32] Yamanari M. Tsuda S. Kokubun T. Shiga Y. Omodaka K. Yokoyama Y. Himori N. Ryu M. Kunimatsu-Sanuki S. Takahashi H. Maruyama K. Kunikata H. Nakazawa T., “Fiber-based polarization-sensitive OCT for birefringence imaging of the anterior eye segment,” Biomed. Opt. Express 6(2), 369–389 (2015). 10.1364/BOE.6.000369 25780730 PMC4354580

[33] Li E. Makita S. Hong Y.-J. Kasaragod D. Yasuno Y., “Three-dimensional multi-contrast imaging of in vivo human skin by Jones matrix optical coherence tomography,” Biomed. Opt. Express 8(3), 1290–1305 (2017). 10.1364/BOE.8.001290 28663829 PMC5480544

[34] Li J. Feroldi F. de Lange J. Daniels J. M. A. Grünberg K. de Boer J. F., “Polarization sensitive optical frequency domain imaging system for endobronchial imaging,” Opt. Express 23(3), 3390–3402 (2015). 10.1364/OE.23.003390 25836196

[35] Villiger M. Zhang E. Z. Nadkarni S. K. Oh W.-Y. Vakoc B. J. Bouma B. E., “Spectral binning for mitigation of polarization mode dispersion artifacts in catheter-based optical frequency domain imaging,” Opt. Express 21(14), 16353–16369 (2013). 10.1364/OE.21.016353 23938487 PMC3724396

[36] Lo W. C. Y. Villiger M. Golberg A. Broelsch G. F. Khan S. Lian C. G. Austen W. G. Jr. Yarmush M. Bouma B. E., “Longitudinal, 3D imaging of collagen remodeling in murine hypertrophic scars *in vivo* using polarization-sensitive optical frequency domain imaging,” J. Invest. Dermatol. 136(1), 84–92 (2016). 10.1038/JID.2015.399 26763427 PMC4809366

[37] Villiger M. Lorenser D. McLaughlin R. A. Quirk B. C. Kirk R. W. Bouma B. E. Sampson D. D., “Deep tissue volume imaging of birefringence through fibre-optic needle probes for the delineation of breast tumour,” Sci. Rep. 6(1), 28771 (2016). 10.1038/srep28771 27364229 PMC4929466

[38] Villiger M. Braaf B. Lippok N. Otsuka K. Nadkarni S. K. Bouma B. E., “Optic axis mapping with catheter-based polarization-sensitive optical coherence tomography,” Optica 5(10), 1329–1337 (2018). 10.1364/OPTICA.5.001329 PMC6581518 31214632

[39] Li Q. Karnowski K. Noble P. B. Cairncross A. James A. Villiger M. Sampson D. D., “Robust reconstruction of local optic axis orientation with fiber-based polarization-sensitive optical coherence tomography,” Biomed. Opt. Express 9(11), 5437–5455 (2018). 10.1364/BOE.9.005437 30460138 PMC6238922

[40] Villiger M. Otsuka K. Karanasos A. Doradla P. Ren J. Lippok N. Shishkov M. Daemen J. Diletti R. van Geuns R. J. Zijlstra F. van Soest G. Libby P. Regar E. Nadkarni S. K. Bouma B. E., “Coronary plaque microstructure and composition modify optical polarization: a new endogenous contrast mechanism for optical frequency domain imaging,” JACC Cardiovasc. Imaging 11(11), 1666–1676 (2018). 10.1016/j.jcmg.2017.09.023 29248662 PMC5994172

[41] Kasaragod D. Fukuda S. Ueno Y. Hoshi S. Oshika T. Yasuno Y., “Objective evaluation of functionality of filtering bleb based on polarization-sensitive optical coherence tomography,” Invest. Ophthalmol. Vis. Sci. 57(4), 2305–2310 (2016). 10.1167/iovs.15-18178 27127929

[42] Spöler F. Kray S. Grychtol P. Hermes B. Bornemann J. Först M. Kurz H., “Simultaneous dual-band ultra-high resolution optical coherence tomography,” Opt. Express 15(17), 10832–10841 (2007). 10.1364/OE.15.010832 19547440

[43] Sacchet D. Moreau J. Georges P. Dubois A., “Simultaneous dual-band ultra-high resolution full-field optical coherence tomography,” Opt. Express 16(24), 19434–19446 (2008). 10.1364/OE.16.019434 19030031

[44] Cimalla P. Walther J. Mehner M. Cuevas M. Koch E., “Simultaneous dual-band optical coherence tomography in the spectral domain for high resolution in vivo imaging,” Opt. Express 17(22), 19486–19500 (2009). 10.1364/OE.17.019486 19997169

[45] Lim Y. Hong Y.-J. Duan L. Yamanari M. Yasuno Y., “Passive component based multifunctional Jones matrix swept source optical coherence tomography for Doppler and polarization imaging,” Opt. Lett. 37(11), 1958–1960 (2012). 10.1364/OL.37.001958 22660086

[46] Baumann B. Choi W. Potsaid B. Huang D. Duker J. S. Fujimoto J. G., “Swept source/Fourier domain polarization sensitive optical coherence tomography with a passive polarization delay unit,” Opt. Express 20(9), 10229–10241 (2012). 10.1364/OE.20.010229 22535114 PMC3366588

[47] Wang Z. Lee H.-C. Ahsen O. O. Lee B. Choi W. Potsaid B. Liu J. Jayaraman V. Cable A. Kraus M. F. Liang K. Hornegger J. Fujimoto J. G., “Depth-encoded all-fiber swept source polarization sensitive OCT,” Biomed. Opt. Express 5(9), 2931–2949 (2014). 10.1364/BOE.5.002931 25401008 PMC4230879

[48] Prestin S. Rothschild S. I. Betz C. S. Kraft M., “Measurement of epithelial thickness within the oral cavity using optical coherence tomography,” Head Neck 34(12), 1777–1781 (2012). 10.1002/hed.22007 22318761

[49] Schindelin J. Arganda-Carreras I. Frise E. Kaynig V. Longair M. Pietzsch T. Preibisch S. Rueden C. Saalfeld S. Schmid B. Tinevez J.-Y. White D. J. Hartenstein V. Eliceiri K. Tomancak P. Cardona A., “Fiji: an open-source platform for biological-image analysis,” Nat. Methods 9(7), 676–682 (2012). 10.1038/nmeth.2019 22743772 PMC3855844

[50] Reichart P. A. van Wyk C. W. Becker J. Schuppan D., “Distribution of procollagen type III, collagen type VI and tenascin in oral submucous fibrosis (OSF),” J. Oral Pathol. Med. 23(9), 394–398 (1994). 10.1111/j.1600-0714.1994.tb00083.x 7529836

[51] Tsai M.-T. Chen Y. Lee C.-Y. Huang B.-H. Trung N. H. Lee Y.-J. Wang Y.-L., “Noninvasive structural and microvascular anatomy of oral mucosae using handheld optical coherence tomography,” Biomed. Opt. Express 8(11), 5001–5012 (2017). 10.1364/BOE.8.005001 29188097 PMC5695947

[52] Wang J. Zheng W. Lin K. Huang Z., “Development of a hybrid Raman spectroscopy and optical coherence tomography technique for real-time in vivo tissue measurements,” Opt. Lett. 41(13), 3045–3048 (2016). 10.1364/OL.41.003045 27367097

[53] Chen P.-H. Wu C.-H. Chen Y.-F. Yeh Y. C. Lin B.-H. Chang K.-W. Lai P.-Y. Hou M.-C. Lu C.-L. Kuo W. C., “Combination of structural and vascular optical coherence tomography for differentiating oral lesions of mice in different carcinogenesis stages,” Biomed. Opt. Express 9(4), 1461–1476 (2018). 10.1364/BOE.9.001461 29675295 PMC5905899

[54] Sawazaki-Calone I. Rangel A. Bueno A. G. Morais C. F. Nagai H. M. Kunz R. P. Souza R. L. Rutkauskis L. Salo T. Almangush A. Coletta R. D., “The prognostic value of histopathological grading systems in oral squamous cell carcinomas,” Oral Dis. 21(6), 755–761 (2015). 10.1111/odi.12343 25825335

[55] Junqueira L. C. U. Cossermelli W. Brentani R., “Differential staining of collagens Type I, II and III by Sirius Red and Polarization microscopy,” Arch. Histol. Jpn. 41(3), 267–274 (1978). 10.1679/aohc1950.41.267 82432

[56] Montes G. S. Krisztán R. M. Shigihara K. M. Tokoro R. Mourão P. A. Junqueira L. C., “Histochemical and morphological characterization of reticular fibers,” Histochemistry 65(2), 131–141 (1980). 10.1007/BF00493161 6153646

